# Analysis of Modern vs. Conventional Development Technologies in Transportation—The Case Study of a Last-Mile Delivery Process

**DOI:** 10.3390/s22249858

**Published:** 2022-12-15

**Authors:** Mariusz Kostrzewski, Yahya Abdelatty, Ahmed Eliwa, Mirosław Nader

**Affiliations:** 1 Faculty of Transport, Warsaw University of Technology, 00-662 Warsaw, Poland; 2 Independent Researcher, 31-323 Cracow, Poland; 3 Doctoral School, Warsaw University of Technology, 00-661 Warsaw, Poland

**Keywords:** conventional delivery trucks and vans, autonomous delivery robots, RADRs, civil delivery drones, UAV

## Abstract

Transportation plays a significant role in the global economy and society and takes part in a lot of different processes such as mass transportation and the supply chain. Therefore, it is crucial to introduce modern technologies in this area of the economy in the context of Industry 4.0. The main scope of this study is to develop a model that supports analyzing last-mile logistics modern solutions using the latest technologies such as road autonomous delivery robots (RADRs), civil drones, or smart bikes, and compare them to conventional solutions (delivery vehicles). Multi-criteria decision analysis (MCDA) was applied to build a formal comparison model that scores the solutions and weights different criteria according to decision-makers and placeholders, to rank the solutions from the most crucial option to the weakest in a predetermined scenario with set parameters and conditions (three varied scenarios were included in the present investigation). The results of the model were in favor of using civil drones or smart bicycles to perform light deliveries in small urban areas (these key findings support the assumptions that are often manifested in speech in the context of the use of new technologies). The modern solutions scored almost 40–80% higher in total in the conglomeration of assessment criteria (such as safety, economy, laws and regulations, operation time for the delivery, environment, and payload) than the conventional solution, which indicates the importance of studying the implementation of such technologies. An interesting result of the study is the operational cost reduction by ca. 60–74% in favor of autonomous delivery robots, 89–93% in favor of civil delivery drones, and 87–90% in favor of smart bikes vs. conventional delivery trucks/vans. Yet, it should be underlined that the results may vary with different assumptions within the MCDA method.

## 1. Introduction

Since ancient times, people have transported goods from one location to another using different means of transport. The economy and society have traditionally placed a high value on transportation, which continues to play a vital role in many applications including mass transit, public and private transportation, and supply chain and logistics procedures. Therefore, it is necessary to implement modern technology in the transportation industry. The more advanced the logistics and transportation systems are, the more advantages they accrue to societies and economies throughout the world. To identify knowledge gaps and development possibilities that keep transportation and logistics in sync with the growth of other industries, it is important to thoroughly study, investigate and assess the introduction of modern technology in the predefined context. This context is presented in Section 1.1., which is the starting point for the literature review given in Section 1.2. Section 1.1. concludes with an outline of the paper’s structure, and Section 1.2. presents the research gaps.

### 1.1. A Sense of the Study

How significant is the advancement of logistics and transportation for humanity? New technologies have been introduced, tested, and utilized to create more dependable, efficient, and environmentally friendly means of transport since the emergence of transportation networks to allow the transfer of people and freight. Logistics and transportation are two different yet closely related terms. While logistics is described as the administration and planning of the manufacturing of freight (logistics management), handling and storing it, and then moving it to the end user (logistics engineering), transportation is the movement of passengers and freight by various branches and modes in a variety of environments including land, water, and air [1]. The definition of the term logistics is not unified, yet logistics as such ties a network of manufacturers, distributors, retailers, and transportation companies. The definition of logistics demonstrates how essential transportation is to its operation as it oversees the overall process of managing how resources are acquired, stored, and transported from the origin to their destination [2]. With so many stakeholders and types of processes, any technological advance, even the slightest, can serve to make significant progress in the transportation and logistics conglomerate.

In an era of massive use of fossil fuels, it is of utmost importance that these new technologies are designed to be powered by alternative energy sources and their operations controlled by appropriate sensors. While this is not the immediate focus of this study, it is also worth considering this aspect in the selection of means of transport applied to transportation and logistics processes. To make this selection rational, it is necessary to analyze which of the means of transport and equipment, developed in recent years, are worthy of further development based on their technological, economic, and financial benefits. As both logistics and transportation are continually enriched with new, supportive technologies, it is necessary to investigate and compare conventional and modern technologies. Consequently, it is obligatory to compare them to each other to evaluate the wisdom of replacing conventional technologies with modern ones. This paper presents an investigation of conventional delivery trucks/vans with modern devices and means of transport equipped with sensors such as autonomous delivery robots, civil delivery drones, and smart bikes.

The structure of the paper is as follows: Firstly, a review of the literature on conventional and modern technologies applied in certain transport processes and operations is finalized with the specification of research gaps. Secondly, the assessment method and three scenarios are briefly described. They are applied to obtain the results given in Section 3, where data are accumulated and converted into proper evaluation criteria. Finally, the obtained results are presented in Section 3 and briefly discussed in Section 4 together with their sensitivity analysis, and the paper is concluded in Section 5.

### 1.2. Literature Review

**Figure 1:**
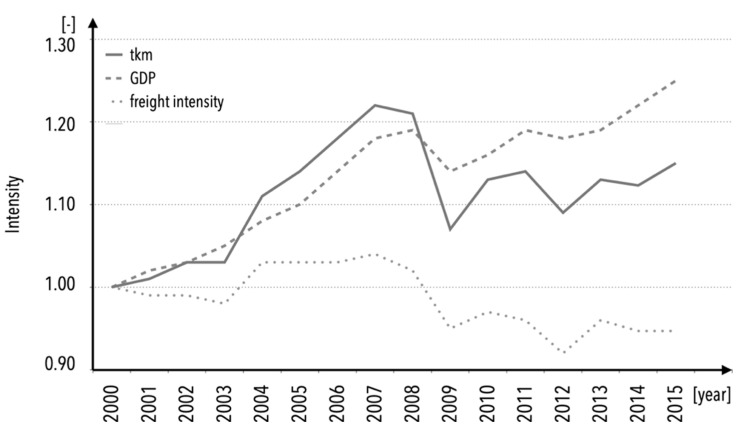
Inland freight transport volumes, based on EEA [3,4].

The growth in demand for freight transportation over the past few decades is what gives the development of transportation and logistics such significance in the context of modern technologies application. The European Environment Agency (EEA) reports that since 2000, the demand for freight transportation has increased by 14% for road transportation and by about 11.5% for rail transportation [3,4] in 2015 (it is worth mentioning that these websites were archived with the attribution of discontinued indicators [5]). Figure 1 depicts the development of this demand as well as the impact of significant economic occurrences such as the 2008 financial crisis, in which enormous credit swaps were mispriced and caused significant businesses and economies to file for bankruptcy [6].

Germany, one of the top nations for manufacturing, coined the phrase “Industrie 4.0” [7], which paves the way for the development of new technologies with applicability across many industries. Consequently, manufacturing, logistics, and transportation systems are significantly impacted by technologies such as cyber-physical systems (CPS) and the Internet of Things (IoT). Industry 4.0 is one issue that has contributed to the displacement of conventional technologies by modern ones, not only in manufacturing but also in other economic spheres, such as transportation and logistics. The American Society of Mechanical Engineers (ASME) [8] foreordained modern technologies in transportation which are of interest in the current transportation and logistics systems, namely smart bicycles, maglev trains, multi-directional elevators, next-generation lithium-ion batteries, safer autonomous navigation, enhanced drones, and hypersonic air travel to mention solely the most crucial. Among the mentioned modern technologies, there are multiple which have been implemented in freight transport, such as autonomous delivery robots and civil delivery drones.

The logistics sector is one of the most significant businesses that will profit from the usage of autonomous vehicles in its transportation operations. The use of autonomous vehicles for freight transportation can reduce crashes and use 10 to 15% less energy [9,10]. Additionally, since fully autonomous vehicles will not be subjected to work time restrictions, they will save operational and personnel expenses and boost productivity [11]. To identify the necessary adjustments to legislation, infrastructures, and logistics, researchers have started to develop models that assist in predicting the adoption rate of autonomous trucks. According to [12], fully autonomous trucks would lower operational expenses by 45%, or between USD 85 and 125 billion, as 65% of US freight is transported by trucks. These numbers are notable, and by lowering freight charges, they might be very advantageous to any delivery company and its clientele. This also implies that it is crucial to concentrate on and adopt mobile robots and autonomous trucks together with other modern technologies as soon as feasible.

Many delivery or manufacturing companies, particularly those strongly related to logistics, may benefit from the implementation of autonomous vehicles coupled with other modern technologies, investigated in this paper. With the introduction of new technologies in the framework of Industry 4.0, logistics has significantly advanced in recent years. Due to demand from customers who expect prompt and same-day deliveries, novel technologies such as autonomous vehicles and robotics have been the subject of investigation. There are various scenarios that academics and specialists in the logistics sector have presented that serve to make the process of implementing autonomous trucks more understandable. Viscelli presented six scenarios for the use of autonomous trucks in 2018 [13]:

- Platooning: A method of driving in which several trucks employ autonomous capabilities to follow a leading vehicle. Only under specific circumstances do the trailing truck drivers assume control.
- Human–drone platooning: A scenario that closely resembles the first, except that the trailing trucks are driverless autonomous vehicles. With autonomous capabilities that allow them to make judgments in certain situations, the trailing trucks precisely mimic the behaviors of a leading vehicle that is piloted by a person.
- Exit-to-exit autonomous trucks with remote operation: In this scenario, autonomous vehicles are remotely controlled by operation centers in challenging environments such as small cities. However, trucks employ their autonomous technology to move freight unassisted on smooth surfaces such as motorways. This situation may present several difficulties, such as a loss of connections.
- Autopilot scenario: The American Trucking Association (ATA) prefers the scenario in which a driver and a vehicle alternately handle the driving process utilizing autonomous autopilot capabilities that regulate the speed and position of a truck in its lane.
- Exit-to-exit autonomous trucks: In this scenario, level 4 fully autonomous trucks are employed in transportation on interstates or highways without drivers. Only while moving cargo to and from these autonomous vehicles in urban and rural locations do human drivers interfere.
- Facility-to-facility trucking: In this scenario, driverless autonomous trucks handle all aspects of facility-to-facility transportation. Without human assistance, trucks should be able to maneuver through small spaces.

Each of the scenarios has different technological requirements, benefits, and drawbacks since it has a significant influence on businesses and the employment of drivers. Logistics companies should carefully consider each scenario before implementation.

To place the following technologies, it should be mentioned what exactly is an autonomous vehicle. A vehicle that uses modern technology to adapt to its environment and make the same decisions as a human driver is known as an autonomous (self-driven, driverless, or robotic) vehicle. When a robotic vehicle is listed, it is worth mentioning that an autonomous robot (autonomous delivery robot) is designed and engineered to operate autonomously in its surroundings and for lengthy periods without human intervention. It must not only carry out its delivery mission well but also assesses the numerous circumstances that are changing around it and behave accordingly [14]. A certain applicability of robots in internal and external freight transport was developed in [15]. The inevitable application of such technology is enhanced by shortened travel time and decreasing cost, efficiencies that usage of autonomous delivery devices (autonomous delivery robots) can assure in comparison to traditional van deliveries [16]. The delivery costs for both solutions were investigated in [16], and the authors found that although autonomous delivery robots are more competitive vs. conventional vans their range is shorter and their storage capacity is limited.

Autonomous robotic systems have the potential to significantly improve operational safety and efficiency. Because of the explosive expansion of e-commerce, providing quicker, more inexpensive, and sustainable last-mile delivery becomes increasingly vital. Many challenges, such as reduced capacity, driver shortages, damaged and stolen merchandise, unsuccessful delivery efforts, increased traffic congestion, and so on, can be handled with autonomous delivery robots (autonomous delivery devices) [14].

On the other hand, unmanned aerial vehicles (UAVs) that may be used for civil purposes such as freight delivery are known as civil delivery drones. With the evolution of technology and the right programming, drones may be fully autonomous and accomplish tasks without human involvement. Drones employ sensors and technologies that enable human control. Civil drones were first used in the logistics industry to support the control of inventory in warehouses [17,18,19]. However, due to their many benefits, organizations are now researching the usage of drones for last-mile deliveries [20,21,22,23,24,25,26,27] in various European cities [21], and especially in Berlin [23], Milan [24], and Rotterdam [26]. Other transportation-related uses for drones include traffic and infrastructure monitoring [28].

Drone delivery is also becoming more popular in industries such as healthcare, food, postal service, and shipping. The ability of drones to fly into rural or otherwise inaccessible places more readily and quickly than other modes of transportation is perhaps their most significant advantage for healthcare delivery (therefore this technology can contribute to leveling off freight transport poverty) [29]. Another significant advantage of using drones in the healthcare business is that they may carry medicinal supplies to locations that are inaccessible by other modes of transportation. Drones can travel to locations where vehicles and motorcycles cannot. Drones are utilized to deliver medical items such as vaccinations, drugs, blood, and even kidneys in numerous locations [29]. The challenge in the application of drone solutions is, for example, a payload connected with a cable to a drone—this problem is considered e.g., in [30] based on cargo transportation by quadcopters (quadrotors).

Recently, typical coupling of technology consists of truck-based/van-based drone delivery systems such as the examples presented in [31,32]. To follow this concept, it is worth mentioning the comparison between drones and track deliveries which was of interest in [33]. The results proved that package deliveries by drones may be cost-effective compared to conventional trucks as more packages are loaded per tour; however, drones supported by a truck ensure the lowest cost-related indicators such as total cost and cost per delivery (the same would be related to smart bicycles as well). Also in [34], the authors proposed a delivery system that consists of a drone and public urban transportation vehicles (trains, trams, public buses, etc.).

With the usage of drones, same-day delivery and environmentally friendly transportation will be very much viable and more prevalent in the nearest future. Since drones move in the air, delivery would not be hampered by difficult terrain or heavy traffic. Additionally, drones will be able to function without direct surveillance of people, properly delivering goods to the end user, owing to technologies such as the Internet of Things, machine learning, and location sensors.

Some concerns have been voiced about the usage of drone delivery services, especially the necessity to relocate or establish additional distribution centers closer to customers [35]. A recent patent application [36] by Amazon Technology Inc. for a fulfillment center designed to accommodate the landing and take-off of UAVs in densely populated areas (from here on referred to as drone beehives) appears to confirm the industry is giving this delivery alternative more serious consideration [21].

To address some of the present urban logistical challenges, cargo bikes have been recommended as an alternative to diesel vehicles for parcel delivery. These bikes are known as smart bicycles and are fully equipped with a set of sensors [37]. Additionally, remote sensing technology is increasingly being applied in smart bicycles together with cyclists’ smartphones (for example, the authors of [38] presented a sensor board and accompanying software for bicycle controlling; additionally the authors of a review paper [39] mentioned that most smartphones are the main inspiration for smart bicycles—the authors of [40] referred directly to the Internet of Things technology in this context). The authors of [41] emphasized that cargo bikes are only used for very particular types of deliveries, primarily small packages. The use of delivery vans and cargo bikes has primarily been studied in operations research. Cargo bikes have the following advantages over motorized delivery vehicles: they are smaller; therefore, they can ride more easily through narrow streets and find parking locations faster and closer to the recipient. They are electric-assisted vehicles (or even unmanned ones [42]); therefore, they produce less noise or direct greenhouse gas emissions, while vehicle purchase and maintenance costs are lower, and labor costs are about the same. Cargo bikes, on the other hand, have substantially less capacity and their batteries limit their range. Cargo bikes may cause driver fatigue, and their top speed is often lower than that of conventional vans [43].

The authors of [44] presented a study consisting of delivery scenarios involving over-the-road vehicles, sidewalk automated delivery robots, UAVs, and smart bicycles (also known as e-cargo bikes) for US conditions. Different autonomous technologies for urban last-mile logistics operations were developed in [45] to optimize costs and externalities. The authors proved that in numerous regions characterized by low-density service, truck-launched delivery drones can decrease the range of total operations costs by almost 25%. Meanwhile, delivery robots would reduce such costs by 60% in more dense urban environments. Other optimization results are given by the mentioned authors in their very detailed research report.

When last-mile logistics of small-goods deliveries is considered, bicycles cannot be omitted, especially smart bicycles. These vehicles are being equipped with collision-free warning systems, mostly applied by using accelerometers/gyroscopes, LIDAR, sensors, and networking communication, as the authors of a recent review paper reported [39]. Such systems significantly affect cyclists’ and small goods’ safety.

Despite the popularity of novel technologies’ considerations and applications in logistics and transportation, the authors have not found any in-depth study in the research literature that would provide a comparison of the aforementioned technologies with those conventionally applied in the delivery process. Some exceptions can be observed in the popular science press [46] (yet also in this case there is a lack of quantitative comparative indicators) and research papers [16,33,44,47] (with different goals and assumptions in making comparisons than in the case of this article). Thus, the authors consider the lack of such a comparison to be a research gap. This research gap raises a lot of questions from company owners, users/customers, etc. Are these new technologies supporting the companies in satisfying customer needs? Are they helping the companies deliver products/packages in more efficient way? How much of a difference does it make? Therefore, this paper compares a selection of conventional and modern technologies used in transportation and logistics.

The above-mentioned questions and juxtaposition of technologically diverse solutions led the authors to consider their key research objectives. Firstly, the assessment criteria for a comparison of the aforementioned technologies must be carefully matched. Some of the typically applied criteria can be either quantitative or qualitative. In the case of the second type of criteria, a support in the form of expert evaluation is necessary. Consequently, the opinion on a particular criterion is influenced by such and expert’s consideration, and therefore, it is worth considering how experts’ opinions influence any results. To fill the research gap, by comparing conventional and modern technologies with quantitative indicators, and addressing the other aspects mentioned in this paragraph, the methodology presented in the following section was applied.

## 2. Materials and Methods

A formal model (mathematical model) was developed to assess the technological expansion of modern solutions applied in transportation and logistics and compare them with conventional solutions. The model is based on a decision theory, specifically, multi-criteria decision analysis (MCDA; also: multiple-criteria decision-making—MCDM), to give a score for the solutions based on different criteria [48,49,50]. The MCDA method is extended by the concept of environmental, social, and corporate governance (ESG) [51]. ESG-related criteria are an inclusive part of the model. It should be underlined that although the MCDA method has been well known for years and represents one of the research trends prior to Industry 4.0, it continuously attracts researchers’ interest in the area of Industry 4.0 assessment as e.g., in [52,53,54,55]. Safety, economy, laws and regulations, time for delivery, environment, and payload were the selected criteria for this study. This set of criteria is uniquely selected by the authors and has been hardly applied in any other study.

The solutions used as input technologies to the model were civil drones, road autonomous delivery robots (RADRs), and smart bicycles, as modern types of technologies applied in the novel delivery process. These solutions use modern technology development in the context of Industry 4.0 and Logistics 4.0. Moreover, a traditional technology such as conventional vans applied for the delivery process (as a conventional type of technology application) was the final solution implemented in the model to compare it to the modern solutions in terms of how differently they operate in freight transport. The authors assumed a delivery process occurring in urban areas in the case of any assessed technology.

Apart from MCDA application in the study, the scenario technique was applied as well [56,57]. The scenario technique involves a holistic description of a studied system (model), detailing the most complete possible relevant factors affecting a system (model), and consequently obtaining realistic courses of decision-making situations. For an accurate implementation of the model, it is good practice to simulate real-life conditions in creating a scenario to produce more reliable outcomes. The scenario also allows the authors to evaluate results and draw indicative conclusions that may support future enhancements of the model. It should also be mentioned that a scenario provides the necessary variables which solve any obstacle that arises regarding the comparison of different sets of data during the solutions comparison.

The scenarios used for the implementation of the model have the purpose of finding out the best solution to be applied in last-mile logistics [58,59,60]. Last-mile logistics plays an important role in the operations related to the supply chain; therefore, researching and developing solutions that use modern technologies could lead to much more efficient supply chain operations. The scenarios are characterized by the following settings:

- Logistics processes of delivery are investigated for a small urban area.
- The US laws and regulations are applied in the scenarios.
- Using American manufacturers of modern technologies.
- The logistics processes have the purpose of delivering 10 or 20 small packages at a time with an average weight of 1.5 kgs per package and traveling 16 km or 45 km.

The MCDA uses a set of criteria to score solutions based on the mentioned scenarios. The weight of each criterion depends significantly on the decision-makers (four independent experts investigated and assessed the presented technologies) according to the criterion’s importance, and therefore the scenarios were required to test the model. Moreover, as experts’ opinions may be case-specific, it is worth developing a sensitivity analysis depending on experts’ opinions (it is given in Section 4). Different objective and subjective weighting methods have been developed to be used in such situations [61]. The use of more than one weighting method in the model at a time can indicate the accuracy and consistency of the results. Therefore, this model used two weighting methods, which are the point allocation method (allocating points to criteria according to priority) and the rank sum method (criteria ranked according to importance, then its weight is calculated). In view of the MCDA-based application, it is worth enriching the considerations with the following research questions:

- RQ1: Which of the preliminary defined optimization criteria (given in Section 3) support the most decisions related to choosing a means of transport in terms of the presented modern and conventional technologies?
- RQ2: How do the experts’ opinions influence the results of MCDA application?

Scoring the solutions is realized after identifying and weighting the criteria and depends on the data collected and their type. Data collected can be qualitative or quantitative. Qualitative data can pose a challenge when it is evaluated. To overcome this challenge, it can be transformed into numbers using a Likert scale of 1 to 5 where 1 relates to characteristics defined as “poor” and 5 as “excellent” [62]. After using multiple equations to find the scores, these scores were finally used in a spreadsheet created by the authors to identify and compute the required results for the study and help to compare the different modes/technologies of transport.

The next section presents the necessary data and information that are obligatory for assessing the solutions with the use of the abovementioned method(s), and the assessment itself is also developed.

## 3. Results

### 3.1. Identifying Criteria

Criteria that fit the scenarios discussed in Section 2 are identified in the present section. Decision-makers usually identify the most important criteria based on the importance of a process of elimination to come up with the best combination fitted to the scenarios. The criteria used for the model in these scenarios are as follows:

1. Economy: Capital spending for the technologies used in the modern solutions, energy consumption costs, and delivery costs are all expenses that are very important to take into account when comparing modern solutions vs. conventional ones (the symbol of this criterion is K2) [63,64,65,66].
2. Time for delivery: Time taken to deliver packages in last-mile logistics can be critical to the operator and the end user nowadays when competition is rising, and speed is an important pillar to the success of the business (symbol K4) [33,34,67].
3. Payload: The payload of the solution can affect the properties of the packages to be delivered and their amount, and therefore the number of returns the means of transport has to make to the logistics operator (symbol K6) [30,33,68,69].
4. Laws and regulations: Complying with local laws is always a must for all kind of businesses and can be challenging for modern solutions that might have an unclear position in the current laws and regulations (symbol K3) [70,71].
5. Safety: Safety is always a great concern for modern solutions in all industries and is very important to take into consideration (symbol K1) [39,72,73,74].
6. Environment: Last-mile logistics contribute 25–30% of greenhouse gas emissions in the sector of transportation and that creates the need for modern solutions to reduce emissions (symbol K5) [75,76,77,78].

Table 1 shows the ranking of the above criteria according to their importance for the decision-makers and their aim in the model. All the criteria certainly need to be given an underscoring, and the scoring topology is given in the following Section 3.2.

### 3.2. Scoring Solutions

Collecting large amounts of data can be very supportive in the scoring process for the solution as it becomes indicative and informative about the different aspects of modern technologies under discussion in this paper. These data were applied to score modern solutions in this model according to each criterion. Most of the data collected for this model are qualitative since these solutions use modern technologies that are still being under testing and development. The Likert scale mentioned in the methodology is used in this process [62].

The parameters, described in Section 2 for the scenarios, are useful when calculating scores for the solutions regarding their economic aspects, and the formulas (1)–(3) were used to provide results that allow these solutions to be compared.

The first scenario (S1) considers deliveries of 10 packages per day for one year and a distance/package of 16 km. Two more scenarios were developed (all of them were given the same examination as in the case of S1):

- Second scenario (S2)—20 package deliveries per day for one year and a distance per package of 16 km (scenario is set with double the deliveries to be satisfied at the same distance).
- Third scenario (S3)—10 package deliveries per day for one year and a distance per package of 45 km (scenario with a longer distance of delivery and the same number of packages to be delivered as in S1; in this scenario, it was decided to take into account the size of megacities, and it was proposed to consider delivering of parcels at a distance determined by the span of the currently largest megacity in the world, namely the Tokyo Megalopolis Region [79]; the distance was assumed based on [80], where a radius of Tokyo Megalopolis Region area is given as ca. 45 km).

The total cost of each scenario operation is examined with Equation (1).

(1) TCD=((ACP)+(ECM×i))×j×k,

(2) CC=CE+MC,

(3) EC=TCD+CC,

where:

- *TCD—*total cost of delivery;
- *ACP—*average cost per package;
- *ECM—*energy cost per kilometer;
- *i—*distance of a delivery (16 km);
- *j—*number of packages in a delivery (10 packages);
- *k*—total duration of deliveries (365 days);
- *CC—*capital costs;
- *CE—*cost of equipment;
- *MC—*maintenance costs per year;
- *EC—*economy costs.

Table 2, Table 3, Table 4 and Table 5 present the scores given in the cases of S1–S3 to the four solutions: RADRs, civil drones, conventional vans, and smart bicycle. Each criterion is scored, and a description is provided along with references to the data collected. Scores based on qualitative data use the Likert scale, 1 being a qualitative term such as “poor assessment of a criterion in the case of a certain technology” and 5 being a qualitative term such as “excellent assessment of a criterion in the case of a certain technology”, and are given this score based on the how the solutions perform regarding this criterion compared to the other solutions.

The analysis and evaluation of variants characterized by decision variables of different types and units, such as those presented in the discussion so far, requires the unification and normalization of the results. For this purpose, one of the methods of zero standardization was applied. These methods are characterized by the adoption of a certain uniform point of reference [49], which is the gap between the extreme values obtained in each sample (minimum and maximum values of each *k*-criterion given in Equations (4) and (5)). Table 6 and Table 7 show the normalization of the scores given to the three solutions according to the aim of the analysis, whether to maximize or minimize the effects. The normalization is performed using Table 1 and Table 6 and the following formulas:

(4) $$\mathrm{Maximization}:\;K_{norm}=\frac{k-k_{min}}{k_{max}-k_{min}},$$

(5) $$\mathrm{Minimization}:\;k_{norm}=\frac{k_{max}-k}{k_{max}-k_{min}},$$

### 3.3. Weighting Criteria

The solutions for each criterion were given scores after ranking the criteria in accordance with the needs of the business. The decision-makers assigned weights to the criteria based on their requirements. As previously mentioned, there are various approaches to weighting a criterion. The formulas used in both the rank sum method and point distribution method are described in Table 8.

The rank sum and point distribution strategies are applied in this model. The criteria weights are presented in Table 9 and Table 10.

### 3.4. Combining Scores and Weights

To attribute a total score to each analyzed technology and assist the decision-maker in selecting the best course of action, the scores and weights of the various criteria are combined at this stage. Following Equation (6), the weights are multiplied by the scores and then added together. Consequently, a total score is obtained.

(6) $$\overset{K}{\underset{k=1}{\sum }}w_{k}\cdot X_{k}^{1/2/3/4norm}$$

The combination of the scores and weights utilizing both types of weighting criteria are shown in Table 11, Table 12, Table 13 and Table 14 (scenarios S1–S3), which is evidently consistent and produces an acceptable outcome.

## 4. Results Discussion

Methods based on experts’ opinion such as the rank sum or rank allocation method are subjective; therefore, the sensitivity of changes depending on the weights of certain criteria is worth analyzing (it is based on S1 solely). The assumption was made that each successive criterion would be analyzed incrementally, in five-point increments, while keeping the other four criteria constant and topping up the scores to 100 in total for criterion K6 (there was no particular reason for choosing this criterion as a top-up) in correlation to Table 9. This means that if, for example, *K*1 = 10, then, *K*2 = *K*3 = *K*4 = *K*5 = 5 and *K*6 = 70. The values of the weights together with the point method application for general assessment of all the solutions, namely autonomous delivery robots, civil delivery drones, conventional delivery trucks/vans, and smart bicycles are given in Table 15.

**Figure 2:**
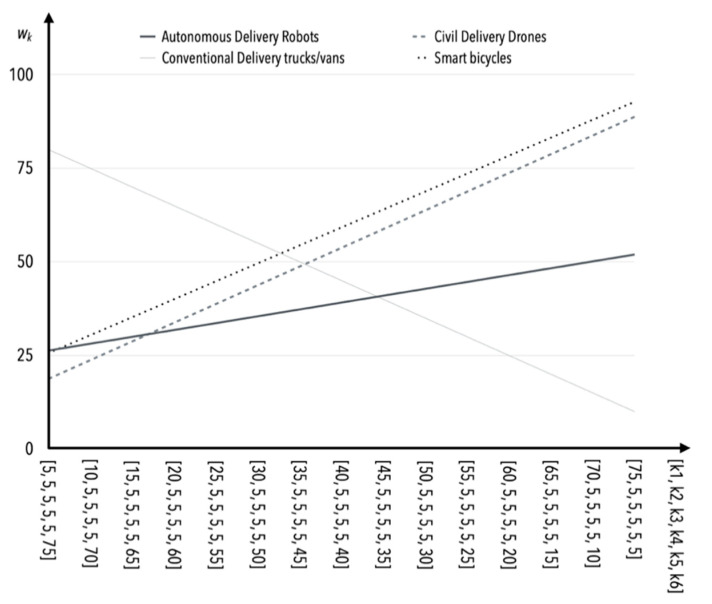
The influence of weight value changes for criterion *K*1 on criteria *K*2*–K*5 (in the case of S1).

**Figure 3:**
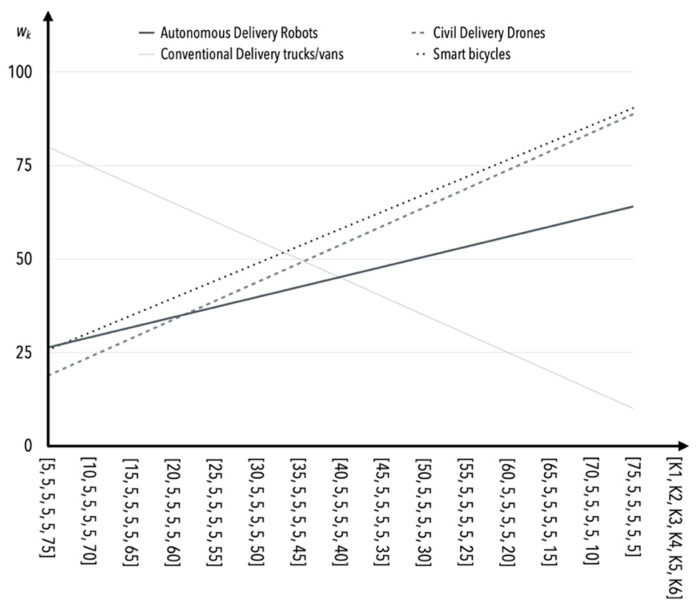
The influence of weight value changes for criterion *K*2 on criteria *K*1, *K*3*–K*5 (in the case of S1).

The greatest susceptibility to change (and consequently sensitivity) occurs as the value of the criterion *K*1 weight increases and the values of the weights of the other criteria (i.e., *K*2–*K*5) remain constant (Table 15, Figure 2). Safety is one of the more important criteria in the opinion of experts, and therefore this behavior of the model is not questionable or surprising. The incremental effect of the values of *K*2 weights results in similar behavior of the model as in the case of *K*1 (Table 15, Figure 3).

**Figure 4:**
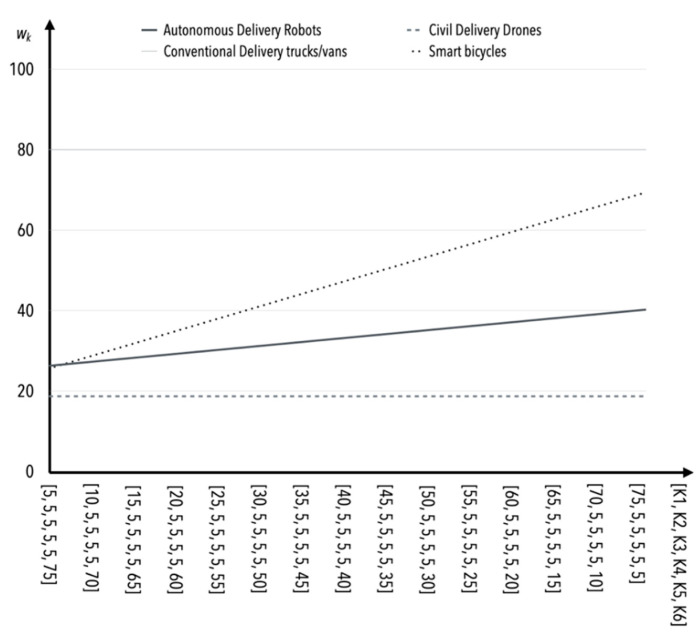
The influence of weight value changes for criterion *K*3 on criteria *K*1*, K*2, *K*4, *K*5 (in the case of S1).

Varying sensitivity to changes in the values of weights for different means of transport occurs for criterion *K*3. Such heterogeneity is primarily related to the uncertainty regarding the legal regulation of modern, unmanned means of transport. However, in practical applications, the high weighting values for this criterion are not expected (Table 15, Figure 4).

**Figure 5:**
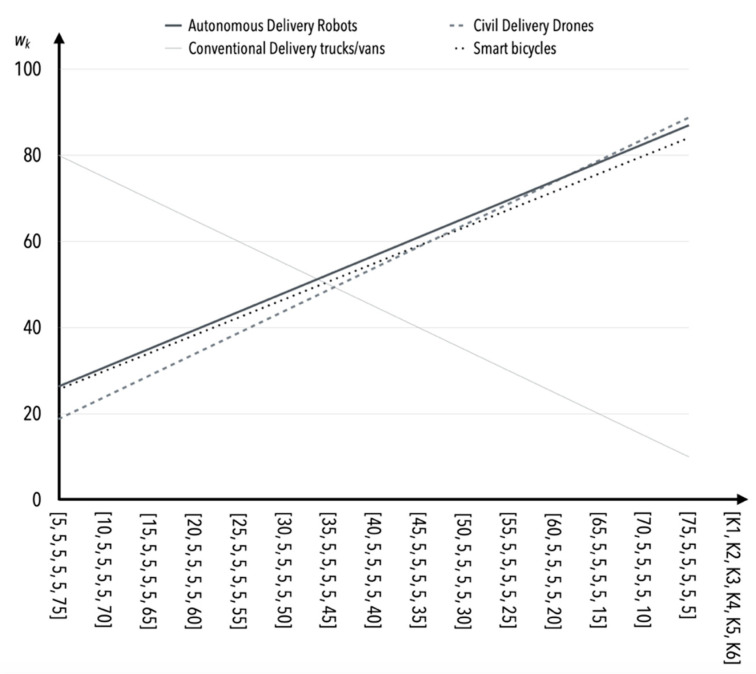
The influence of weight value changes for criterion *K*4 on criteria *K*1–*K*3, *K*5 (in the case of S1).

**Figure 6:**
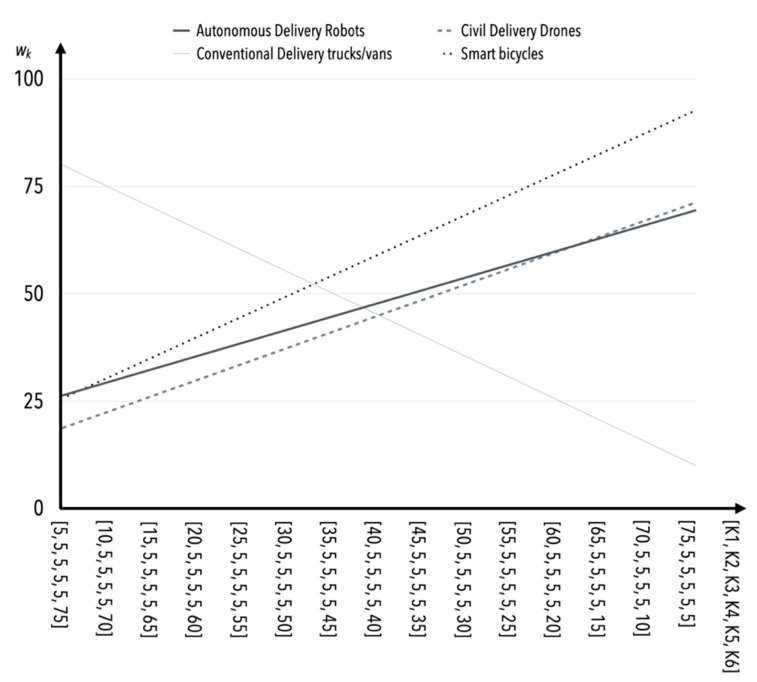
The influence of weight value changes for criterion *K*5 on criteria *K*1*–K*4 (in the case of S1).

The effects of changes in the value of the weighting index for criterion *K*4 are like each other within modern technologies (Figure 5). Thus, this criterion appears to be the least sensitive to changes for modern technologies. Similar effects were observed when analyzing criterion *K*5 (Figure 6).

The MCDA model’s findings made it abundantly evident that contemporary solutions are superior to those that are already in use. Using smart bicycles or civil drones in settings of this type would be the most proper choice, according to the analysis and criterion function of the investigated scenario S1. With a slight difference in overall score, RADRs are allocated in the third position, and conventional trucks and vans take the lowest allocation.

The outcomes demonstrate how the decision-intent makers are represented in the final scores; safety and economy were the most important factors that contributed to these outcomes because they were given the greatest weights. The results of the two weighting systems are shown in Table 16, together with the total scores for each choice and the percentage difference between the present and more advanced solutions.

## 5. Conclusions

Nowadays, it is observable that economic areas are becoming more and more intertwined, blurring the boundaries between them. A similar blurring of boundaries occurs between transportation branches. It turns out that processes previously reserved for one branch of transport, such as road transport, can now be carried out by other branches of transport as well and with higher efficiency. This is owing to technological and engineering development. Some delivery processes, hitherto traditionally reserved for road transport, can be carried out by means of other modes of transport such as air transport with UAV (including civil drones) or with less traditional means of transport such as RADRs.

A simplified mathematical model built on MCDA was developed to evaluate the solutions and compare them, providing logistics companies with the chance to select the most fitting option that would best meet their requirements. It should be underlined that any development of MCDA was not the aim of this study—the importance of this investigation was the selection of data for the method in such a way that the results were meaningful and in line with reality. Moreover, the given set of criteria is unique, and not applied in any other study according to our review of the scientific literature.

The model was then applied to a scenario with predetermined parameters and conditions to determine which of conventional vehicles, civil drones, smart bicycles, and

RADRs would be an effective choice in line with the assumptions and decision-making criteria. According to the model, given the information and criteria specified in the scenarios, the usage of civil drones, autonomous robots, or smart bicycles can be around 40–80% better than that of conventional vehicles (Table 16). More significant differences in the operational effectiveness of using new technologies as opposed to conventional road vehicles (effectiveness not just in economic aspect) occur as the distance increases (i.e., from 60% to 80%; in S1, the distance was 16 km, while in S3 it was 45 km). On the other hand, with an increase in the number of packages to be transported, profitability still occurs although its magnitude is not as spectacular (40–60%). These results back up the findings of [45], which among others stated that delivery robots would reduce operational costs by 60% in more dense urban environments. In the case of actual research, the financial reduction was also equal to ca. 60% in favor of autonomous delivery robots vs. conventional delivery trucks/vans (based on Table 6; the criterion of the economy). It should be also underlined that in the case of scenarios S2 and S3, the financial reduction was even higher i.e., over 70%, whereas the civil delivery drones’ operational cost was reduced by almost 90% compared to conventional delivery trucks/vans in the case of S1, up to over 93% in the case of S3 (based on Table 6; the criterion of the economy). Meanwhile, the smart bicycles’ operational cost was reduced by 87–90% compared to conventional delivery trucks/vans in the case of all the scenarios (based on Table 6; the criterion of the economy). Such comparison in the case of civil drones and smart bicycles was not presented in the previously analyzed publications.

It should be underlined, following [120,121], that dense urban regions can promote non-motorized means of transport such as, for example, smart bicycles, yet at least two conditions should be fulfilled for safe and high-quality streets. Smart bicycle usage can ensure a low-polluting and low-cost transportation alternative (as it was proved in the previous paragraph). It is worth noting that in the context of poverty of transport for cargo mentioned above, the results demonstrate the high potential for the application of the presented solutions to offset or deal with the issue of the inability to deliver goods to hard-to-reach places. This, certainly, requires the necessary practical research work. The conditions mentioned above, together with a weight of safety criterion assumed based on experts’ opinions, support the answer to RQ1, namely the safety criterion supports the most decisions related to choosing a means of transport in terms of the presented modern and conventional technologies. Meanwhile, the answer to RQ2 can be studied based on the given sensitivity analysis, which is why based on the research results both civil drones and smart bicycles can be applied for last-mile logistics with similar positive results in comparison to conventional solutions (as given in Table 16).

However, it should be underlined that in the currently presented assessment, more criteria than costs were incorporated into the model, which is progressive compared with other research results. These results are promising enough that a significant reshaping of the existing business models of delivery companies can be expected soon, which will benefit from the advantages of multimodal transportation, i.e., the transportation of goods, using two or more modes of transport. Moreover, this study proved the validity of the application of modern devices and means of transport such as autonomous delivery robots or civil delivery drones within the transportation and logistics process. As these devices and means of transport are equipped with sensors and actuators, it is worth focusing on their technical development to improve their current abilities even more.

While researching the topic and developing the mathematical model, there were some limitations in terms of resources and data. Since the technologies mentioned in the context of Industry 4.0 and Logistics 4.0 are very recent, there are still limited data and resources providing extensive details and information that covers all aspects related to these technologies. For future research on similar topics, it is advised to collect more data with high accuracy that would provide an output from the model with high reliability; another recommendation would be using other weighting methods for the criteria analysis to make sure the results are consistent. In future research, an analysis based on a wider range of means of transport will be carried out (e.g., e-delivery vehicles, autonomous trolleys, and other types of vehicles will be considered), and at the same time it will be possible to conduct analyses under real conditions—validation of the obtained results will be carried out. At the same time, this research demands varying the scenarios with different determinants in the number of packages, their weight, and alternative distances. Moreover, comparative analysis can also be provided for different domains such as medical supply, Internet of Things, general logistics systems, etc.—nevertheless, this is a complex and very wide-ranging problem worth devoting experimental research to. It is a matter of future research as well.

## Figures and Tables

**Table 1:** Criteria Ranking and Aim.

Symbol	Criteria	Sub-Criteria	Aim
K1	Safety	-	Maximize
K2	Economy	Capital costs/spending	Minimize
		Average delivery	
		Energy costs	
K3	Laws and Regulations	-	Maximize
K4	Time for Delivery	-	Minimize
K5	Environment	-	Maximize
K6	Payload	-	Maximize

**Table 2:** RADRs scores in the case of S1 (unless otherwise mentioned).

$\mathrm{RADRs}—X_{k}^{1}$				
Criteria		Score	Description	References
K1	Safety	3 (scale 1 to 5)	Low weight means fewer dangerous accidents. Robots operate at low speeds. Avoids human error. Reduces death due to crashes by 60%.	[81,82]
K2	Economy	USD 28,995 S2: USD 53,523 S3: USD 33,466	Average cost per pack, *ACP* = USD 5.950 Energy per kilometer, *ECM* = USD 0.035 Average cost of RADR, *CE* = USD 5000 Maintenance, *MC* = USD 1000	[16,83,84]
K3	Laws and Regulations	3 (scale 1 to 5)	National Highway Traffic Safety Administration (NHTSA) updates regulations in USA. High insurance costs are imposed.	[62,85], S2: [86]
K4	Time	15.00 min S2: 16.50 min S3: 45.75 min	The speed of delivery depends on the distance and traffic; this is an average by a manufacturer.	[87], S2: [16]
K5	Environment	4 (Scale 1 to 5)	RADRs have low emissions; however, generating electricity emits greenhouse gases and harmful elements, whose emissions can be calculated according to the source of generating electricity.	[88], S2: [16]
K6	Payload	215.5 kg	Average payload	[89]

**Table 3:** Civil drone scores in the case of S1 (unless otherwise mentioned).

$\mathrm{Civil}\;\mathrm{Delivery}\;\mathrm{Drones}—X_{k}^{2}$				
Criteria		Score	Description	References
K1	Safety	4 (scale 1 to 5)	High-end technology for sensing and avoiding obstacles. Safety precaution systems by some manufacturers, such as releasing threads and deploying parachutes in case of falling. The drones still pose risks of falling on pedestrians and causing injuries.	[81]
K2	Economy	USD 7442 S2: USD 11,160 S3: USD 8247	Average cost per pack, *ACP* =USD 0.8800 Energy per kilometer, *ECM* = USD 0.0063 Average cost of drone, *CE* = USD 2000 Maintenance, *MC* = USD 2000	[90,91]
K3	Laws and Regulations	2 (scale 1 to 5)	Federal Aviation Authority (FAA) started allowing for the testing of drones under certain conditions such as specific altitudes and areas. To be certified in some states of the USA, licensed pilots should be included in the operations.	[90,92],
K4	Time	15 min S2: less than 30 min S3: 85 min	Speed of delivery depends on the distance and flying time; this is an average by manufacturers.	[90,93], S2: [94]
K5	Environment	4 (scale 1 to 5)	Drones have low emissions; however, generating electricity emits greenhouse gases and harmful elements, whose emissions can be calculated according to the source of generating electricity.	[89], S2: [94]
K6	Payload	2.27 kg	Average payload.	[90]

**Table 4:** Delivery vehicle scores in the case of S1 (unless otherwise mentioned).

$\mathrm{Conventional}\;\mathrm{Delivery}\;\mathrm{Trucks}/\mathrm{Vans}—X_{k}^{3}$				
Criteria		Score	Description	References
K1	Safety	2 (scale 1 to 5)	Since 2016, there have been 2180 crashes caused by one delivery service. Delivery vans and trucks are heavy, and their incidents are fatal. Moreover, they lack safety features and technology.	[95]
K2	Economy	USD 73,292 S2: USD 156,320 S3: USD 127,777	Average cost per pack, *ACP* = USD 8.08 Energy per kilometer, *ECM* = USD 0.02 Average cost of van, *CE* = USD 30,000 Maintenance, *MC* = USD 65,000	[96,97,98]
K3	Laws and Regulations	5 (scale 1 to 5)	Laws and regulations are already developed and updated for conventional vehicles.	[81]
K4	Time	240 min S2: within 240 min S3: within 240 min	Speed of delivery depends on the distance and traffic; this is an average deduced from FedEx.	[99]
K5	Environment	1 (scale 1 to 5)	Vans and trucks produce almost 29.4% of the greenhouse gas emissions in the transportation system.	[100]
K6	Payload	1595 kg	Average payload from the data of 5 vans used in delivery.	[101]

**Table 5:** Smart bicycle scores in the case of S1 (unless otherwise mentioned).

$\mathrm{Deliveries}\;\mathrm{by}\;\mathrm{Smart}\;\mathrm{Bicycles}—X_{k}^{4}$				
Criteria		Score	Description	References
K1	Safety	4 (scale 1 to 5)	The number of fatalities involving bicyclists was slightly higher than 2% in 2009 and less than 3% in 2018 [102]. These numbers are expected to be lower when smart bicycles are used. E-assist bikes are often ridden more quickly. Consequently, there can be elevated safety hazards. E-bike incidents can simply be prevented if human error can be reduced. Some researchers, such as the authors of [103], considered helmets that display awareness messages.	[102,103,104]
K2	Economy	USD 9676 S2: USD 15,043 S3: USD 15,372	Average cost per pack, *ACP* = USD 0.56 (based on values of equipment given in [105]) Energy per kilometer, *ECM* = USD 0.05 Average cost of smart bicycle, *CE* = USD 4000 (assessed based on 80% of smart bikes compilation given in [106] Maintenance, *MC* = USD 1115 (based on [106])	[105,106,107,108,109,110]
K3	Laws and Regulations	4 (scale 1 to 5)	Laws and regulations are being developed and updated for smart bikes.	[111,112]
K4	Time	43 min S2: 43 min S3: 120 min	Speed of delivery depends on the distance and traffic; this is an average deduced based on distance and the mean value of bicycle speed based on [113]. According to a new study, e-cargo bikes make deliveries 60% faster than delivery vehicles in urban areas.	[41,108,113,114]
K5	Environment	5 (scale 1 to 5)	Smart bicycles emit greenhouse gases as well as other vehicle types in the transportation system. However, these emissions are significantly lower in comparison with other vehicle types. This is for example due to the fact that a smart bike battery is only 1–2% of the size of an electric car battery (reduced energy consumption) [115]. The authors of [115] suggested that CO2 emissions would be reduced by 15 million tons yearly if everyone used bikes.	[108,114,115,116]
K6	Payload	150 kg	Average payload including a person.	[108,114,117,118,119]

**Table 6:** Before normalizing scores in the case of all the scenarios (unless otherwise mentioned).

Solution/ Criteria	$\mathrm{Autonomous}\;\mathrm{Delivery}\;\mathrm{Robots},\;X_{k}^{1}$	$\mathrm{Civil}\;\mathrm{Delivery}\;\mathrm{Drones},\;X_{k}^{2}$	$\mathrm{Conventional}\;\mathrm{Delivery}\;\mathrm{Trucks}/\mathrm{Vans},\;X_{k}^{3}$	$\mathrm{Smart}\;\mathrm{Bicycles},\;X_{k}^{4}$
Safety	3	4	2	4
Economy	S1: 28,995 S2: 53,523 S3: 33,466	S1: 7442 S2: 11,160 S3: 8247	S1: 73,292 S2: USD 156,320 S3: USD 127,777	S1: 9676 S2: USD 15,043 S3: USD 15,372
Laws and Regulations	3	2	5	4
Time for Delivery	S1: 15.00 S2: 16.50 S3: 45.75	S1: 15.00 S2: 30.00 S3: 85.00	240.00	S1, S2: 43.00 S3: 120.00
Environment	4	4	1	5
Payload	215.5	2.27	1595	150

**Table 7:** After normalizing scores in the case of all the scenarios.

Solution/ Criteria	$\mathrm{Autonomous}\;\mathrm{Delivery}\;\mathrm{Robots},\;X_{k}^{1norm}$			$\mathrm{Civil}\;\mathrm{Delivery}\;\mathrm{Drones},\;X_{k}^{2norm}$			$\mathrm{Conventional}\;\mathrm{Delivery}\;\mathrm{Trucks}/\mathrm{Vans},\;X_{k}^{3norm}$			$\mathrm{Smart}\;\mathrm{Bicycles},\;X_{k}^{4norm}$		
	S1	S2	S3	S1	S2	S3	S1	S2	S3	S1	S2	S3
Safety	0.50	0.50	0.50	1.00	1.00	1.00	0.00	0.00	0.00	1.00	1.00	1.00
Economy	0.67	0.71	0.79	1.00	1.00	1.00	0.00	0.00	0.00	0.97	0.97	0.94
Laws and Regulations	0.33	0.33	0.33	0.00	0.00	0.00	1.00	1.00	1.00	0.67	0.67	0.67
Time for Delivery	1.00	1.00	1.00	1.00	0.00	0.80	0.00	1.00	0.00	0.88	1.00	0.62
Environment	0.75	0.75	0.75	0.75	0.75	0.75	0.00	0.00	0.00	1.00	1.00	1.00
Payload	0.13	0.13	0.13	0.00	0.00	0.00	1.00	1.00	1.00	0.04	0.04	0.04

**Table 8:** Weighting methods to be used in the model, where *k*—consecutive number of criteria, *w_k_*_—_weight of the *k*-th criterion, *N*—total number of criteria, *R_k_*—rank of the *k*-th criterion.

Weighting Method	Point Allocation Method	Rank Sum Method
Formula	Total weight of criteria is equal to 100	$w_{k}=\frac{N-R_{k}+1}{\sum_{k=1}^{N}N-R_{k}+1}$

**Table 9:** Point method for weighting criteria.

Method	Point Allocation Method
How is it used?	100 points are allocated for the weights
Criteria	Weight *w_k_*
Safety	35
Economy	20
Laws and Regulations	15
Time for Delivery	15
Environment	10
Payload	5

**Table 10:** Rank sum method for weighting criteria.

Method	Rank Sum Method		
How Is It Used?	Rank Sum Formula Section 3.2		
Criteria	Rank	Weight *w_k_*	Norm. weight
Safety	1	6	0.286
Economy	2	5	0.238
Laws and Regulations	3	4	0.190
Time for Delivery	4	3	0.143
Environment	5	2	0.095
Payload	6	1	0.048

**Table 11:** Total scores, point allocation method.

Weight *w_k_*	Solution/Criteria	Autonomous Delivery Robots			Civil Delivery Drones			Conventional Delivery Trucks/Vans			Smart Bicycles		
		S1	S2	S3	S1	S2	S3	S1	S2	S3	S1	S2	S3
35	Safety	17.50	17.50	17.50	35.00	35.00	35.00	0.00	0.00	0.00	35.00	35.00	35.00
20	Economy	13.45	14.16	15.78	20.00	20.00	20.00	0.00	0.00	0.00	19.32	19.47	18.81
15	Laws and Regulations	5.00	5.00	5.00	0.00	0.00	0.00	15.00	15.00	15.00	10.00	10.00	10.00
15	Time for Delivery	15.00	15.00	15.00	15.00	0.000	11.97	0.00	14.98	0.00	13.13	15.00	9.27
10	Environment	7.50	7.50	7.50	7.50	7.50	7.50	0.00	0.00	0.00	10.00	10.00	10.00
5	Payload	0.67	0.67	0.67	0.00	0.00	0.00	5.00	5.00	5.00	0.21	0.21	0.21
Total		59.00	60.00	61.00	77.50	62.50	74.47	20.00	34.98	20.00	87.66	89.67	83.28

**Table 12:** Total scores, rank sum method—in the case of S1.

Weight	Solution/Criteria	Autonomous Delivery Robots	Civil Delivery Drones	Conventional Delivery Trucks/Vans	Smart Bicycles
0.286	Safety	0.1429	0.2857	0.0000	0.2857
0.238	Economy	0.1602	0.2381	0.0000	0.2300
0.190	Laws and Regulations	0.0635	0.0000	0.1905	0.1270
0.143	Time for Delivery	0.1429	0.1429	0.0000	0.1251
0.095	Environment	0.0714	0.0714	0.0000	0.0952
0.048	Payload	0.0064	0.0000	0.0476	0.0020
Total		0.5872	0.7381	0.2381	0.8650

**Table 13:** Total scores, rank sum method—in the case of S2.

Weight	Solution/Criteria	Autonomous Delivery Robots	Civil Delivery Drones	Conventional Delivery Trucks/Vans	Smart Bicycles
0.286	Safety	0.1429	0.2857	0.0000	0.2857
0.238	Economy	0.1686	0.2381	0.0000	0.2317
0.190	Laws and Regulations	0.0635	0.0000	0.1905	0.1270
0.143	Time for Delivery	0.1429	0.0000	0.1427	0.1428
0.095	Environment	0.0714	0.0714	0.0000	0.0952
0.048	Payload	0.0064	0.0000	0.0476	0.0020
Total		0.5956	0.5952	0.3807	0.8845

**Table 14:** Total scores, rank sum method—in the case of S3.

Weight	Solution/Criteria	Autonomous Delivery Robots	Civil Delivery Drones	Conventional Delivery Trucks/Vans	Smart Bicycles
0.286	Safety	0.1429	0.2857	0.0000	0.2857
0.238	Economy	0.1879	0.2381	0.0000	0.2239
0.190	Laws and Regulations	0.0635	0.0000	0.1905	0.1270
0.143	Time for Delivery	0.1429	0.1140	0.0000	0.0883
0.095	Environment	0.0714	0.0714	0.0000	0.0952
0.048	Payload	0.0064	0.0000	0.0476	0.0020
Total		0.6149	0.7092	0.2381	0.8221

**Table 15:** Sensitivity analysis in the case of S1 (where CDD—civil delivery drones, T/V—conventional delivery trucks/vans, SB—smart bicycles).

**Criteria**	**k1**	5	10	15	20	25	30	35	40	45	50	55	60	65	70	75
	**k2**	5	5	5	5	5	5	5	5	5	5	5	5	5	5	5
	**k3**	5	5	5	5	5	5	5	5	5	5	5	5	5	5	5
	**k4**	5	5	5	5	5	5	5	5	5	5	5	5	5	5	5
	**k5**	5	5	5	5	5	5	5	5	5	5	5	5	5	5	5
	**k6**	75	70	65	60	55	50	45	40	35	30	25	20	15	10	5
**Vehicle Type**	**RADR**	26.32	28.15	29.98	31.81	33.64	35.47	37.30	39.13	40.97	42.80	44.63	46.46	48.29	50.12	51.95
	**CDD**	18.75	23.75	28.75	33.75	38.75	43.75	48.75	53.75	58.75	63.75	68.75	73.75	78.75	83.75	88.75
	**T/V**	80.00	75.00	70.00	65.00	60.00	55.00	50.00	45.00	40.00	35.00	30.00	25.00	20.00	15.00	10.00
	**SB**	25.64	30.43	35.22	40.02	44.81	49.61	54.40	59.19	63.99	68.78	73.57	78.37	83.16	87.95	92.75
**Criteria**	**k1**	5	5	5	5	5	5	5	5	5	5	5	5	5	5	5
	**k2**	5	10	15	20	25	30	35	40	45	50	55	60	65	70	75
	**k3**	5	5	5	5	5	5	5	5	5	5	5	5	5	5	5
	**k4**	5	5	5	5	5	5	5	5	5	5	5	5	5	5	5
	**k5**	5	5	5	5	5	5	5	5	5	5	5	5	5	5	5
	**k6**	75	70	65	60	55	50	45	40	35	30	25	20	15	10	5
**Vehicle Type**	**RADR**	26.32	29.01	31.71	34.40	37.10	39.79	42.48	45.18	47.87	50.57	53.26	55.96	58.65	61.34	64.04
	**CDD**	18.75	23.75	28.75	33.75	38.75	43.75	48.75	53.75	58.75	63.75	68.75	73.75	78.75	83.75	88.75
	**T/V**	80	75	70	65	60	55	50	45	40	35	30	25	20	15	10
	**SB**	25.64	30.26	34.89	39.51	44.13	48.76	53.38	58.01	62.63	67.25	71.88	76.50	81.12	85.75	90.37
**Criteria**	**k1**	5	5	5	5	5	5	5	5	5	5	5	5	5	5	5
	**k2**	5	5	5	5	5	5	5	5	5	5	5	5	5	5	5
	**k3**	5	10	15	20	25	30	35	40	45	50	55	60	65	70	75
	**k4**	5	5	5	5	5	5	5	5	5	5	5	5	5	5	5
	**k5**	5	5	5	5	5	5	5	5	5	5	5	5	5	5	5
	**k6**	75	70	65	60	55	50	45	40	35	30	25	20	15	10	5
**Vehicle Type**	**RADR**	26.32	27.32	28.31	29.31	30.31	31.31	32.30	33.30	34.30	35.30	36.29	37.29	38.29	39.29	40.28
	**CDD**	18.75	18.75	18.75	18.75	18.75	18.75	18.75	18.75	18.75	18.75	18.75	18.75	18.75	18.75	18.75
	**T/V**	80	80	80	80	80	80	80	80	80	80	80	80	80	80	80
	**SB**	25.64	28.77	31.89	35.02	38.15	41.27	44.40	47.53	50.65	53.78	56.91	60.03	63.16	66.29	69.41
**Criteria**	**k1**	5	5	5	5	5	5	5	5	5	5	5	5	5	5	5
	**k2**	5	5	5	5	5	5	5	5	5	5	5	5	5	5	5
	**k3**	5	5	5	5	5	5	5	5	5	5	5	5	5	5	5
	**k4**	5	10	15	20	25	30	35	40	45	50	55	60	65	70	75
	**k5**	5	5	5	5	5	5	5	5	5	5	5	5	5	5	5
	**k6**	75	70	65	60	55	50	45	40	35	30	25	20	15	10	5
**Vehicle type**	**RADR**	26.32	30.65	34.98	39.31	43.64	47.97	52.30	56.63	60.97	65.30	69.63	73.96	78.29	82.62	86.95
	**CDD**	18.75	23.75	28.75	33.75	38.75	43.75	48.75	53.75	58.75	63.75	68.75	73.75	78.75	83.75	88.75
	**T/V**	80	75	70	65	60	55	50	45	40	35	30	25	20	15	10
	**SB**	25.64	29.81	33.98	38.15	42.32	46.50	50.67	54.84	59.01	63.18	67.35	71.52	75.69	79.87	84.04
**Criteria**	**k1**	5	5	5	5	5	5	5	5	5	5	5	5	5	5	5
	**k2**	5	5	5	5	5	5	5	5	5	5	5	5	5	5	5
	**k3**	5	5	5	5	5	5	5	5	5	5	5	5	5	5	5
	**k4**	5	5	5	5	5	5	5	5	5	5	5	5	5	5	5
	**k5**	5	10	15	20	25	30	35	40	45	50	55	60	65	70	75
	**k6**	75	70	65	60	55	50	45	40	35	30	25	20	15	10	5
**Vehicle Type**	**RADR**	26.32	29.40	32.48	35.56	38.64	41.72	44.80	47.88	50.97	54.05	57.13	60.21	63.29	66.37	69.45
	**CDD**	18.75	22.50	26.25	30.00	33.75	37.50	41.25	45.00	48.75	52.50	56.25	60.00	63.75	67.50	71.25
	**T/V**	80	75	70	65	60	55	50	45	40	35	30	25	20	15	10
	**SB**	25.64	30.43	35.23	40.02	44.81	49.61	54.40	59.19	63.99	68.78	73.57	78.37	83.16	87.95	92.75

**Table 16:** Total scores for solutions.

Method	Trucks/Vans	Civil Drones		Autonomous Robots		Smart Bicycles	
-	Score	Score	% Higher	Score	% Higher	Score	% Higher
S1: Point S2: S3:	20.00 34.98 20.00	77.50 62.50 74.47	74.2% 44.0% 73.4%	59.00 60.00 61.00	64.2% 41.7% 67.2%	87.66 89.67 83.28	77.2% 61.0% 76.0%
S1: Rank S2: S3:	0.2381 0.3807 0.2381	0.7381 0.5952 0.7092	67.8% 36.4% 66.4%	0.5872 0.5956 0.6149	59.5% 59.4% 61.3%	0.8650 0.8845 0.8221	72.5% 57.0% 71.0%

## Data Availability

Not applicable.
